# Advances in HIV Research Using Mass Cytometry

**DOI:** 10.1007/s11904-023-00649-x

**Published:** 2023-01-23

**Authors:** Ashley F. George, Nadia R. Roan

**Affiliations:** 1 Gladstone Institute of Virology, Gladstone Institutes, San Francisco, CA 94158 USA; 2 Department of Urology, University of California at San Francisco, San Francisco, CA 94143 USA

**Keywords:** HIV-1, CyTOF, PP-SLIDE, Viral remodeling, T cells, NK cells

## Abstract

**Purpose of Review:**

This review describes how advances in CyTOF and high-dimensional analysis methods have furthered our understanding of HIV transmission, pathogenesis, persistence, and immunity.

**Recent Findings:**

CyTOF has generated important insight on several aspects of HIV biology: (1) the differences between cells permissive to productive vs. latent HIV infection, and the HIV-induced remodeling of infected cells; (2) factors that contribute to the persistence of the long-term HIV reservoir, in both blood and tissues; and (3) the impact of HIV on the immune system, in the context of both uncontrolled and controlled infection.

**Summary:**

CyTOF and high-dimensional analysis tools have enabled in-depth assessment of specific host antigens remodeled by HIV, and have revealed insights into the features of HIV-infected cells enabling them to survive and persist, and of the immune cells that can respond to and potentially control HIV replication. CyTOF and other related high-dimensional phenotyping approaches remain powerful tools for translational research, and applied HIV to cohort studies can inform on mechanisms of HIV pathogenesis and persistence, and potentially identify biomarkers for viral eradication or control.

## Introduction

Flow cytometry is a staple in HIV research, and has led to important insights in our understanding of HIV transmission, pathogenesis, persistence, and control. However, a major restriction of conventional flow cytometry is the limited number of parameters that can be simultaneously monitored, due to the spectral overlap of fluorophores. To overcome these limitations, about 10 years ago, fluorescent antibodies were substituted with antibodies tagged with heavy-metal isotopes. Coupled with a mass-spectrometry-based readout, the resulting approach—called mass cytometry [1–3], or cytometry by time-of-flight (CyTOF)—enables simultaneous phenotyping of ~ 40 antigens, much more than allowed for by conventional flow cytometry. Additionally, in contrast to other high-parameter single-cell phenotyping approaches using sequencing-based readouts such as single-cell RNAseq (scRNAseq) and cellular indexing of transcriptomes and epitopes by sequencing (CITE-seq), CyTOF is more high-throughput and cost effective. Millions of cells can be readily analyzed by CyTOF in a single run at a cost of several hundred dollars per sample, in contrast to the typically tens of thousands of cells per sample by scRNAseq/CITE-seq which also come with higher costs associated with sequencing. Other advantages of CyTOF are that it can be applied on paraformaldehyde-fixed cells, and enables intracellular protein quantitation [4, 5••, 6•, 7••, 8].

**Fig. 1:**
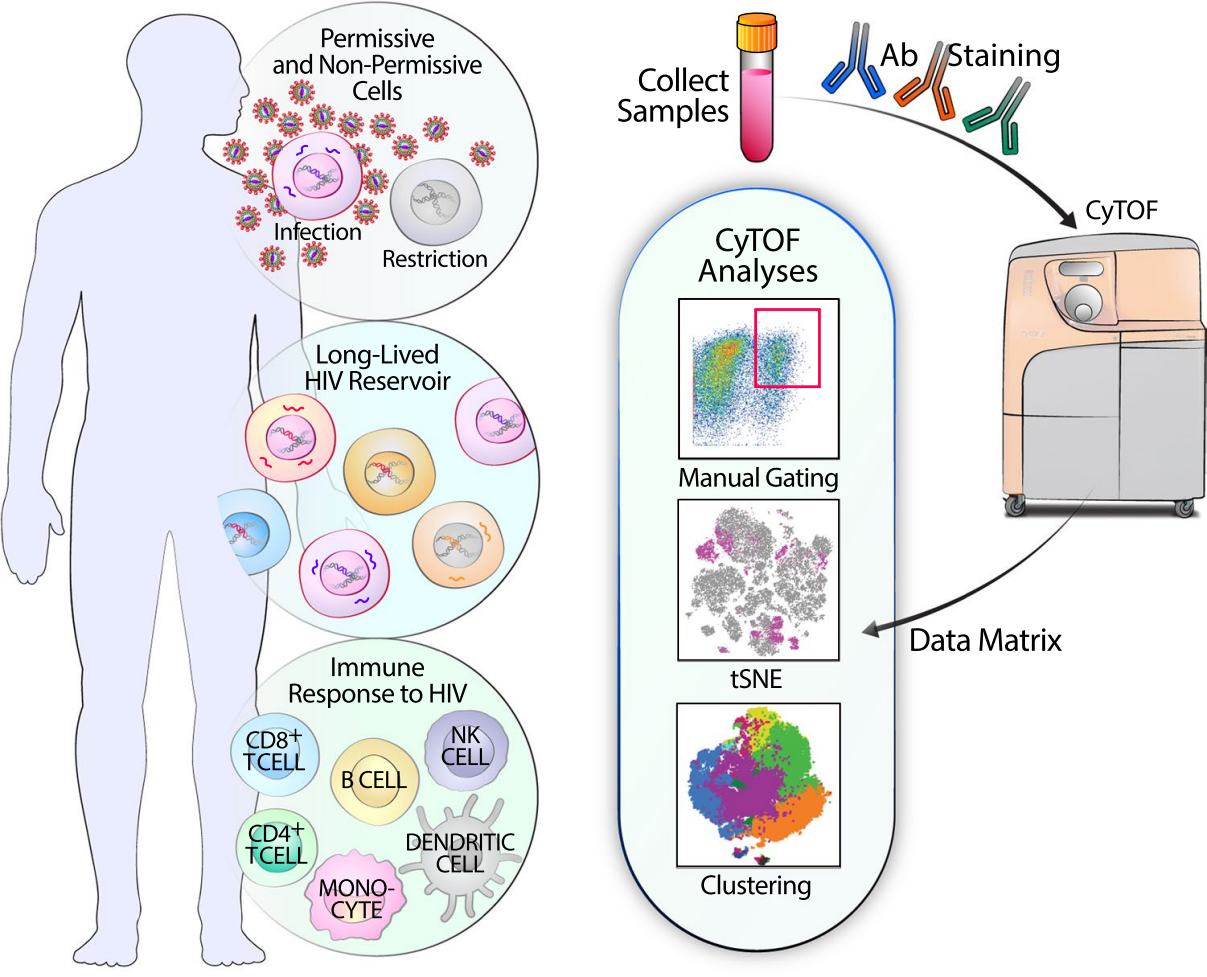
Application of CyTOF for HIV research. CyTOF phenotyping of immune cells has furthered our understanding of HIV-permissive and non-permissive cells, the persistence of the long-term HIV reservoir, and perturbations of the immune system by HIV (left). After blood and tissue specimen collection, isolated cells are stained with antibodies and analyzed using a CyTOF instrument, and the resulting data matrix output is used for further analyses, including manual gating, high-dimensional data visualization, and clustering (right)

CyTOF has allowed for multiple single-cell maps to be established in HIV research, including “atlases” of HIV-permissive and non-permissive cells, and has also been used to characterize effector cells that can control HIV infection. These studies have highlighted the vast diversity of what was previously considered as uniform subsets of HIV-permissive cells and immune effectors that can recognize HIV-infected cells (Fig. 1). Furthermore, trajectory or “pseudo-time” analyses of CyTOF data have revealed phenotypic changes caused by HIV-induced remodeling. This review details these advances and discusses future applications of CyTOF in HIV research and beyond.

## Determinants of Cell Permissiveness to HIV Infection

Although CD4 + T cells are primary targets for HIV infection, not all subsets of these cells are equally susceptible to HIV. Multiple studies have implemented CyTOF to define the features of CD4 + T cells most and least susceptible to infection [5••, 6•, 9••, 10–12] (Fig. 1).

### HIV Fusion

One of the earliest CyTOF studies in HIV research examined which CD4 + T cell subsets in tissues HIV enters, or “fuses” to, using an ex vivo model of HIV infection comprised of lymphoid tissue cells obtained from tonsillectomies [9••]. Two major advantages of this model are that (1) a large number of HIV-permissive cells can be isolated from a single tonsillectomy and (2) these cells are naturally permissive to HIV infection in the absence of mitogens, unlike PBMCs, which are poorly susceptible in the absence of ex vivo stimulation. CyTOF applied to HIV-fused tonsil cells revealed that CCR5-tropic HIV was capable of entering a broad spectrum of lymphoid tissue memory (Tm) cells but for the most part was unable to fuse to naïve CD4 + T cells. Among CD4 + Tm cells, highest fusion levels occurred in cells with phenotypic features of Th2, Th17, and regulatory T (Treg) cells [9••].

### Using PP-SLIDE to Identify the CD4 + T Cell Subsets Permissive to HIV Infection

In contrast to studying HIV fusion, analysis of productive infection is complicated by the fact that the process results in cellular changes. HIV, and in fact most viruses, remodel the cells they infect [6•, 7••, 9••, 13, 14], making it difficult to determine whether markers differentially expressed on HIV-infected cells are actual markers of HIV-susceptible cells, or merely reflect post-infection remodeling. A classic example of this is the downregulation of cell-surface CD4; HIV-infected cells express low levels of cell-surface CD4 not because HIV preferentially selects CD4-negative cells for infection, but because it downregulates CD4 expression at the cell surface through its accessory proteins Nef and Vpu [13, 14].

The high-dimensional nature of CyTOF phenotyping enabled an approach to distinguish permissivity markers from remodeled antigens. This approach combines CyTOF phenotyping and computational approaches into a pipeline called predicted precursor as determined by single-cell linkage for distance estimation (PP-SLIDE) [6•, 7••, 9••]. PP-SLIDE is based on the concept that despite HIV-induced remodeling, enough of the original phenotype of each cell is retained after infection in a manner that can be captured by high-dimensional CyTOF phenotyping (but not by low-parameter phenotyping by conventional flow cytometry). PP-SLIDE compares each productively-infected CD4 + T cell against an entire “atlas” of uninfected CD4 + T cells from the same donor. Through a k-nearest neighbor (kNN) approach, each productively-infected cell is matched to its most similar cell in the uninfected sample. This most similar cell is considered to harbor the phenotype of the productively-infected cell prior to remodeling, and referred to as a “predicted precursor” cell. Comparing predicted precursor cells to the other atlas cells highlights features of cells permissive or resistant to infection, while comparing predicted precursor cells to infected cells pinpoints proteins that were remodeled as a result of infection [9••]. The sections below describe the use of PP-SLIDE to identify features of HIV-susceptible and resistant cells, and how these PP-SLIDE findings were validated.

### HIV-Permissive CD4 + T Cells

The first PP-SLIDE study was used to define the features of tonsillar CD4 + T cells most susceptible to productive infection [9••]. It revealed productive infection preferentially occurring in Th17 and T follicular helper (Tfh) subsets, including those expressing CD57. Interestingly, RNAscope studies suggested that CD57 + T cells are also preferential targets of HIV infection in vivo in lymph nodes of viremic individuals [9••].

While tonsil cells were a useful model for the characterization of HIV-permissive cells derived from tissue, they are less relevant for understanding the early events of sexual HIV transmission, which requires the analysis of genital or gut mucosal tissues. Accordingly, PP-SLIDE was subsequently implemented using CD4 + T cells isolated from the female reproductive tract (FRT) [6•]. Strikingly, endometrial CD4 + T cells were more than ten times more susceptible to HIV infection than tonsillar cells, in the absence of mitogen stimulation [6•]. Preferential CD4 + T cell targets from the FRT included many CD4 + T cell subsets: Th1, Th2, Tfh, T resident memory (Trm), and T effector memory (Tem) cells. These subsets, however, exhibited phenotypic differences from their less-susceptible blood counterparts. For example, endometrial Tem cells expressed higher levels of the HIV co-receptor CCR5 and the activation markers CD38 and PD1 than their blood counterparts did, suggesting an increased state of activation. The pro-survival factor BIRC5 was also elevated on HIV-susceptible endometrial Tem cells as compared to blood, suggesting that these cells can serve as a good host for productive HIV infection in part by successfully surviving cytotoxic insult induced by the virus through inhibition of apoptotic pathways [15, 16].

How relevant are the in vitro PP-SLIDE findings to in vivo HIV infection? When CyTOF/PP-SLIDE was applied to in vivo infected cells from viremic individuals, some phenotypic features were found to be shared between in vitro and in vivo HIV-susceptible cells [12]. In particular, a subset of CD29-expressing Tem-like CD4 + T cells was identified in both systems*.* That being said, there were also phenotypic features unique to each of the systems. For example, Th17 cells and memory CD4 + T cells expressing high levels of α4β1 were preferentially targeted for productive HIV infection in vivo, while Tem, transitional memory (Ttm), and Th1/Th17 memory CD4 + T cell subsets were favored in vitro [12].

Although PP-SLIDE only *predicts* which markers are differentially expressed on HIV-susceptible or -resistant cells, numerous studies have validated these predictions through cell sorting experiments [5••, 6•, 7••, 9••, 12]. For example, PP-SLIDE predicted that CD4 + Tm cells expressing low/medium levels of CCR7, CD62L, and CD57, and medium/high levels of CD29 and CD69 were highly susceptible to HIV infection both in vitro and in vivo [12]. To validate this prediction, cells with this expression profile were isolated using multiparameter sorting, exposed to a CCR5-tropic reporter HIV strain and monitored for infection rates. The sorted cells were significantly more susceptible to infection than were total blood CD4 + Tm cells, validating the original predictions made by PP-SLIDE. In another study, PP-SLIDE analysis of HIV-infected cells analyzed by CyTOF-Lec, which simultaneously phenotypes cells for proteins and different glycan structures [5••], revealed that CD4 + Tm cells expressing higher cell-surface levels of the glycan sialic acid are preferentially susceptible to HIV. These findings were similarly validated through sorting experiments which demonstrated that expression levels of sialic acid on CD4 + Tm cells directly correlated with the susceptibility of the cells to HIV infection [5••].

### HIV-Induced Remodeling

Understanding how HIV remodels host cells after infection provides a better understanding of the mechanisms of HIV pathogenesis and persistence. As mentioned earlier, a classic example of HIV remodeling benefiting the virus is the HIV-mediated downregulation of cell-surface CD4 [17]. CD4 downregulation is observed in infected CD4 + T cells from blood, tonsil, and the endometrium [6•, 9••], and confers several advantages to the virus, including enhanced virion infectivity [18] and release [14], and prevention of superinfection [19] and of cell death by antibody-dependent cellular cytotoxicity [20].

CyTOF/PP-SLIDE identified additional HIV-remodeled factors, with implications for HIV transmission and pathogenesis [6•, 9••]. For example, upon infection of tonsillar T cells, HIV downregulated the co-stimulatory molecules CD28 and OX40, which would interfere with T cell signaling [9••]. Additionally, in endometrial T cells, HIV downregulated multiple components associated with signaling through the T cell receptor (CD28, ICOS and CD45RO), which may dampen antigen-specific T cell responses against HIV [6•]. By contrast, in these same cells, HIV upregulated the pro-survival protein BIRC5, the chemokine receptors CCR7 and CXCR5, and the tissue-residency marker CD69. These host factors may promote the survival [16] and migration [9••, 21, 22] of these cells to lymph node follicles where HIV-permissive cells are abundant. HIV also upregulates the glycans sialic acid and fucose on the surface of infected cells [5••]. As sialylated glycans can suppress effector cell immune responses [23] and fucosylation facilitates lymphocyte trafficking [24], HIV remodeling of glycans may help infected cells avoid detection by the immune system while disseminating systemically.

### HIV-Induced Cell Death

CyTOF/PP-SLIDE has enabled not only detailed analyses of productively infected cells, but also studies of cells that do not undergo productive infection, including cells that are preferentially killed by HIV. By assessing what cells are absent at the end of infection while accounting for infection-related remodeling by PP-SLIDE, it was discovered that some subsets of CD4 + Tm cells that are relatively poorly susceptible to productive infection are surprisingly highly susceptible to CXCR4-tropic HIV-induced cell death [11]. These cells can be defined by high expression of the HIV co-receptor CXCR4, a Tcm phenotype (CCR7 + CD62L +), and low expression of checkpoint molecules (PD1, CTLA4) and activation markers (CD69, CD25, HLADR) [11]. By contrast, cells that are resistant to HIV-induced cell death feature low expression of CXCR4, a Tem phenotype (CCR7-CD62L-), and high expression of checkpoint/activation markers (PD1, CTLA4, CD69, CD25, and HLADR). These data suggest that the cells most susceptible to HIV infection are not necessarily the ones most susceptible to HIV-induced cell death, and provide insights into mechanisms underlying abortive or bystander killing by HIV. They also provide an example of how deep phenotyping for CyTOF can be implemented for understanding HIV pathogenesis.

## The Long-Term HIV Reservoir

Cells spared from productive infection include not only those that are preferentially killed by HIV, but also those preferentially targeted for latent infection. These cells are highly relevant for understanding HIV persistence and are a main barrier to a cure for HIV/AIDS. Because latently-infected cells do not express viral proteins, they are difficult to target therapeutically, or to purify for experimental studies. Hence, much research has attempted to identify host proteins that mark cells harboring latent HIV. CyTOF has contributed important insights into the nature of these cells (Fig. 1).

### Using PP-SLIDE to Identify Latency-Prone CD4 + T Cells

Studies using CyTOF/PP-SLIDE had identified a population of highly HIV-fusogenic tissue-derived CD4 + Tm cells expressing high levels of CD127, the alpha chain of the IL7 receptor [9••]. These cells were resistant to productive infection by CCR5-trophic HIV, but preferentially supported latent infection, defined as infection where HIV DNA integration occurs but LTR-driven reporter protein expression is lacking [9••, 25]. Interestingly, these cells appear to be in a quiescent transcriptional state as characterized by low levels of NF-kB and NFAT signaling, but were not completely transcriptionally silent at the HIV LTR as they produced incomplete HIV transcripts [25]. These results are in line with the notion that HIV reservoir cells include those that actively drive transcription of the LTR [26].

### Application of PP-SLIDE to characterize HIV reservoir cells in vivo

CD127 + Tm cells were discovered somewhat serendipitously as a latency-prone cell type, through in vitro studies aimed at understanding how these cells could support HIV-1 fusion but not productive infection. PP-SLIDE, however, can also be used to directly characterize latently-infected cells from people living with HIV (PLWH) [7••]. This is achieved by stimulating cells from ART-suppressed PLWH to reactivate latent HIV, while leaving unstimulated a fraction of cells from the same specimen to generate the patient-specific “atlas” of cells. After analyzing both fractions by CyTOF, PP-SLIDE is used to identify, for reach reactivated cell, the phenotypically most similar cell in the unstimulated atlas. Analogous to the in vitro PP-SLIDE studies, PP-SLIDE analysis of in vivo reservoir cells is based on the assumption that a reactivated latently-infected cell retains enough of its original identity to be classified correctly among the pool of unstimulated atlas cells. The PP-SLIDE identified “predicted precursor” cell thereby harbors the predicted phenotypic features of the original reservoir cell prior to reactivation. Proof-of-concept experiments applying PP-SLIDE to the J-Lat cell line model of HIV latency demonstrated that different reactivated J-Lat clones could be mapped back to their correct precursor cell line with a high degree of accuracy (~ 99.2% of the time) [7••]. The following section describes how PP-SLIDE was applied on in vivo reservoir cells and validated.

### Reservoir Cells from PLWH Exhibit Shared Phenotypic Features

Application of PP-SLIDE on leukaphereses specimens from ART-suppressed PLWH revealed conserved features of inducible reservoir cells between individuals [7••]. These reservoir cells expressed higher levels of markers of T cell activation (CD69, CD25, and HLADR), T cell differentiation state (Tbet, CRTH2, and CCR6), and T cell exhaustion (PD1 and CTLA4), as compared to total memory CD4 + T cells. Interestingly, however, the exhaustion marker TIGIT was preferentially expressed on reservoir cells only in a subset of PLWH. These findings demonstrate that the HIV reservoir is not randomly distributed among CD4 + Tm cells, but instead exists in discrete clusters of cells that share phenotypic features across individuals. This was validated experimentally through the design and implementation of a “universal” sorting panel, which when applied to cells from previously unanalyzed PLWH, could enrich for cells harboring replication-competent HIV-1. Tailored sorting could also markedly enrich for genome-intact proviruses, to the extent where up to 65.2% of all detected proviruses in a sorted population were fully intact (as compared to typically < 1% in total CD4 + T cells from PLWH, since most infected cells harbor defective proviral genomes) as determined by near full-length proviral sequencing [7••]. Therefore, PP-SLIDE is not only capable of defining the phenotypes of unstimulated HIV reservoir cells from PLWH, but also provides a means to enrich for genome-intact, replication-competent HIV reservoir cells from ART-suppressed PLWH.

Of note, PP-SLIDE of in vivo reservoir cells has also been applied on cells isolated from human tissues, where the majority of HIV-infected cells persists [27]. These studies have revealed unique features of tissue-derived reservoir cells distinguishing them from their blood counterparts [7••]. These include high expression of the Trm cell marker CD69 and low expression of the central memory (Tcm) marker CD27 on reservoir cells from the gut as compared to blood. Interestingly, a comparison of reservoir cells from different tissue compartments revealed shared features. For example, reservoir cells from both gut and lymph nodes expressed high levels of CD69 and PD1, suggesting that dual targeting of PD1 and CD69 may be an approach to simultaneously target HIV reservoir cells from multiple tissues.

Therefore, data generated from CyTOF are consistent with the notion that HIV reservoir cells are not a simple, homogenous compartment, but at the same time have revealed common patterns enabling these cells to be enriched for experimental studies, and potentially eventually directly targeted for viral eradication. In future reservoir studies, it will be of value to leverage CyTOF and PP-SLIDE to compare persisting infected cells between different tissue compartments, and to assess sex-based differences in reservoir features which can be impacted by sex steroids [28, 29]. These studies should expand to the analysis of not only phenotypes but also the glycan features of reservoir cells, which may inform on mechanisms of persistence [24]. From a practical standpoint, the ability of CyTOF/PP-SLIDE to identify surface markers that can enrich for replication-competent reservoir cells [7••] can also be leveraged to enrich for these cells by multiparameter sorting, which can allow for sequencing-based analyses of these cells in a more cost-effective manner.

## Immune Cells in the Context of Uncontrolled and Controlled HIV Infection

CyTOF has been valuable not only for defining the phenotypic features of HIV-infected cells, but also for characterizing other immune cells, including effector cells capable of recognizing these infected cells. This is because CyTOF can simultaneously profile all the major subsets of immune cells in a single specimen, or alternatively be used to delve deeply into the phenotypic features of any one particular subset. CyTOF has been used by multiple groups to better understand immunity to HIV (Fig. 1). In particular, CyTOF panels have been developed to analyze T cells [4, 30–32, 33•, 34, 35], monocytes [36], conventional dendritic cells (cDCs) [36], plasmacytoid dendritic cells (pDCs) [36], and natural killer (NK) cells [4, 37–39, 40••] in the context of multiple aspects of HIV infection: during active viremia, during ART, under rare instances of natural control, and in the context of highly exposed seronegatives (HESN) which may undergo abortive HIV infection.

### Viremic Individuals Living With HIV

Uncontrolled viremia during acute and chronic infection leads to immune dysregulation, and CyTOF has been useful to characterize such dysregulation. CyTOF analysis of T cells demonstrated a clonally expanded and dysfunctional subset of CD4 + Tfh cells in the germinal centers of lymph nodes of viremic individuals, as compared to HIV-individuals [34]. These cells were associated with altered B cell distributions within lymph nodes, suggesting inadequate CD4 + Tfh cell support. Viremic PLWH also have more CD69 + CCR7- Trm CD8 + T cells in their lymph nodes as compared to HIV-individuals, and these cells exhibit an effector-like phenotype [33•]; to what extent these cells limit viremia or conversely are dysfunctional requires further studies.

CyTOF has also revealed dysregulation in the DC cell compartments of viremic PLWH. Individuals with high plasma HIV RNA levels had fewer cDCs and pDCs in their blood relative to HIV-individuals [36]. This reduction of DCs is likely a consequence of DCs trafficking to lymphoid tissues in order to prime T cell responses against HIV. This priming, however, may be compromised as these DCs exhibit increased expression of the LILR inhibitory receptors, which would attenuate their ability to properly present antigen and activate T cells [36]. Viremic individuals also harbored increased numbers of a subset of monocytes expressing high levels of MHC class I antigens (HLA-A,B,C), which presents peptides from viral proteins to CD8 + T cells to activate their cytolytic activity [41], and CD64 (FcγRI), an activating Fc receptor which enhances antigen presentation of viral epitopes on the MHC class I molecules [42]. Although these aspects suggest increased antigen presentation capabilities, this same monocytic subset also expressed high levels of the inhibitory receptors LILRB4 and LILRA2, the expression of which may inhibit monocyte activation [43] and CD64-mediated phagocytosis [44], respectively [36]. Together, these data identify multiple dysregulated subsets of immune cells in viremic individuals.

### ART-Suppressed Individuals Living with HIV

CyTOF has also uncovered immune features associated with HIV reservoir size and treatment conditions in ART-suppressed PLWH. The frequency and expression of the signaling molecule CD45 on CD4 + and CD8 + T cells were found to be positively associated with HIV reservoir size in the blood of ART-suppressed PLWH [30]. These CD45^high^ T cells expressed high levels of the activation markers CD38 and HLADR and the immune checkpoint protein PD-1, suggesting that these cells may be exhausted in response to the larger HIV reservoir [30]. Conversely, ART-suppressed individuals with increased cell-surface expression of the NK cell ligand HLA-Bw6 on CD4 + T cells, CD8 + T cells, and monocytes were more likely to have lower levels of cell-associated HIV DNA and RNA, and plasma HIV RNA [38]. Interestingly, individuals with a homozygous HLA-Bw6/6 genotype (HLA-Bw6 on both alleles) are more likely to become post-treatment controllers, who can maintain sustained virologic suppression for months to years following discontinuation of ART [45]. Consistent with the notion of HLA-Bw6-associated HIV control, individuals heterozygous for HLA-Bw4/6 had the lowest levels of cell-associated HIV DNA and RNA, and plasma HIV RNA [38].

Interestingly, immune cell features can also differ between uninfected vs. ART-suppressed individuals, and in the latter depending on when ART was initiated. For example, CyTOF studies demonstrated that blood NK cells are phenotypically altered in chronically treated PLWH as compared to healthy controls, although these differences did not impact the HIV-specific NK cell response upon stimulation ex vivo [46]. In contrast to those who started ART later during chronic infection, HIV + individuals who started ART during acute infection are known to have lower levels of T cell activation [47], a better preserved immune response [48–50], and a smaller HIV reservoir which decays more rapidly [51, 52]. CyTOF analysis has identified subsets of NK and T cells enriched in individuals who initiated treatment during acute as compared to chronic infection. In particular, acutely treated PLWH harbored higher frequencies of a subset of blood-derived CD56-CD16 + NK cells [4], which are also found in PLWH that produce broadly neutralizing antibodies (bnAbs) against HIV and which have been implicated in HIV control [53]. Analysis of specimens from fine needle aspirates of lymph nodes of these individuals has also revealed higher numbers of lymphoid CD4 + Tfh expressing the homeostatic proliferation marker CD127 in the acute group, suggesting that early treatment with HIV can preserve long-lived Tfh responses in the tissue compartment [4]. Future studies should compare the immune features of acute- vs. chronic-treated individuals within mucosal tissues, where HIV initiates infection and which likely harbors a large proportion of the reservoir during ART suppression [27]. Sampling of multiple mucosal tissues will be important in such studies, as CyTOF analysis of FRT and gut specimens from ART-suppressed PLWH revealed phenotypic differences among the T cell compartment between the two sites [8]. In conclusion, CyTOF studies have suggested that initiation of ART during the acute phase of infection can better preserve NK and T cells associated with viral control, and has highlighted the importance of sampling multiple tissue compartments.

### HIV Controllers

Immune features of rare individuals who can maintain viral suppression in the absence of ART have also been characterized by CyTOF. These include elite controllers, who naturally control HIV infection in the absence of ART, and have been classically defined by the expression of protective HLA alleles (HLA-B*57 or *27) which elicit protective CD8 + T cell responses [54, 55]. Interestingly, CyTOF has revealed that elite controllers also harbor higher frequencies of a subset of CD1c + cDCs that express high levels of the inhibitory receptor CD32b and the MHC class II receptor HLA-DR [36]. Compared to other cDCs, these cDCs have enhanced effector functions and are highly efficient at cytokine secretion and induction of naïve T cell proliferation, and may therefore help promote differentiation of protective CD8 + T cell responses associated with elite control [56].

### NK Cells and Resistance to HIV Infection

Immune-mediated control of HIV can occur not only after HIV infection as in elite controllers, but potentially even prevent infection from taking hold in the first place. In two studies by the lab of Catherine Blish, CyTOF-identified features of NK cells were found to associate with protection against HIV acquisition. In the first study, CyTOF was conducted on NK cells from a cohort of women with matched HIV exposure risk [40••]. NK cell diversity was calculated using the Inverse Simpson Index based on the positive expression of 16 NK cell receptors [57]. This analysis revealed that women with low NK cell diversity were less likely to become infected with HIV, despite having equal behavioral risk [40••]. The mechanisms underlying this phenomenon are attributed to NK cell diversity increasing upon terminal differentiation, leading to decreased flexibility of future effector responses. In the second study, CyTOF and the analysis package CytoGLMM [58••] identified protective NK cell features in HESN women, of who despite repeated HIV exposures remained seronegative [37]. Compared to uninfected controls, HESN women were enriched for NK cells with increased expression of the activating NK cell receptors NKp30, NKG2A, and LILRB1 [37], suggesting a heightened cytotoxic potential. Indeed, NK cells from HESN women exhibited increased antibody-dependent cellular cytotoxicity (ADCC) activity, which correlated with increased CD16 expression [37]. Altogether, these CyTOF studies demonstrate that HIV-exposed individuals that remain uninfected are enriched for specific NK cell features, suggesting a potentially important role for NK cells in preventing HIV acquisition.

## Conclusions and Future Directions

In summary, CyTOF and high-dimensional analysis tools have provided insights into HIV cellular transmission, pathogenesis, persistence, and immunity (Fig. 1). As CyTOF can be applied on paraformaldehyde-fixed cells [4, 5••, 6•, 7••, 8], it provides a convenient and powerful tool to characterize infected and uninfected immune cells from tissues, which typically cryopreserve poorly. This can include cells from tissues of non-human primate and humanized mouse models of HIV, as well as a variety of human tissues. The latter includes biopsies from the gut, genital, and lymph nodes [4, 5••, 6•, 7••, 8] of PLWH, as well as additional tissues accessible only post-mortem. End-of-life cohorts, such as Last Gift where terminally ill volunteers with HIV provide prior consent to tissue donation at the time of death [59], have provided important insights into HIV persistence within deep tissues such as the brain. These and other similar cohorts should be leveraged for deep immunophenotyping by CyTOF and related technologies.

Another aspect of HIV biology which has not yet been sufficiently studied that can benefit from CyTOF are studies to understand the determinants of HIV rebound and control following treatment interruption. Such studies require specimens from analytical treatment interruption trials, which may or may not include cure-based interventions [60–63]. As viral rebound is associated with plasmacytoid DC and type I interferon (IFN) responses [64, 65], specialized CyTOF panels designed specifically to interrogate sensors and signal transducers leading to interferon-stimulated genes (ISGs) would be of interest. Of note, CyTOF should be applied not only to study HIV-infected and total immune cells as detailed in this review, but also to delve deeply into the phenotypic and functional features of HIV-specific T cells, as recently performed to characterize antigen-specific T cells in the context of COVID-19 [66–69]. Such studies, particularly when applied to elite or post-treatment controllers, could lead to a better understanding of mechanisms underlying immune-mediated control of HIV.

## Data Availability

Data sharing not applicable to this article as no datasets were generated or analysed.
